# Lifetime revision risk for medial unicompartmental knee replacement is lower than expected

**DOI:** 10.1007/s00167-020-05863-3

**Published:** 2020-02-12

**Authors:** J. A. Kennedy, E. Burn, H. R. Mohammad, S. J. Mellon, A. Judge, D. W. Murray

**Affiliations:** 1 Nuffield Department of Orthopaedics, Rheumatology, and Musculoskeletal Sciences (NDORMS), Botnar Research Centre, University of Oxford, Old Road, Oxford, OX3 7LD UK; 2 Centre for Statistics in Medicine, University of Oxford, Oxford, UK; 3 Musculoskeletal Research Unit, University of Bristol, Bristol, UK; 4 Nuffield Orthopaedic Centre, Oxford University Hospitals NHS Foundation Trust, Oxford, UK

**Keywords:** Lifetime revision risk, Unicompartmental knee replacement, Arthroplasty, Complications

## Abstract

**Purpose:**

Unicompartmental knee replacement (UKR) is widely considered to be a pre-total knee replacement (TKR) particularly in the young. The implication of this is that it is sensible to do a UKR, even though it will be revised at some stage, as it will delay the need for a TKR. The chance of a UKR being revised during a patient’s life time has not previously been calculated. The aim of this study was to estimate this lifetime revision risks for patients of different ages undergoing UKR.

**Methods:**

Calculations were based on data from a designer series of 1000 medial Oxford UKR with mean 10-year follow up. These UKR were implanted for the recommended indications using the recommended surgical technique. Parametric survival models were developed for patients of different ages based on observed data, and were extrapolated using a Markov model to estimate lifetime revision risk.

**Results:**

The estimated lifetime revision risk reduced with increasing age at surgery. Lifetime revision risk at age 55 was 15% (95% CI 12–19), at 65 it was 11% (8–13), at 75 it was 7% (5–9), and at 85 it was 4% (3–5).

**Conclusion:**

Provided UKR is used appropriately, the lifetime revision risk is markedly lower than expected. UKR should be considered to be a definitive knee replacement rather than a Pre-TKR even in the young. These lifetime estimates, alongside established benefits for UKR in speed of recovery, morbidity, mortality and function, can be discussed with appropriate patients when considering whether to implant a UKR or TKR.

**Level of evidence:**

III.

## Introduction

Clear communication of risks and benefits of proposed interventions are required for patients to make educated decisions, and to provide informed consent. Patients tend to have difficulty interpreting revision rates. The chance of having a revision during their lifetime is a simpler notion that is likely to be preferred by patients when communicating risk [9]. Lifetime revision risk can be expected to be highly dependent on an individual’s age at surgery. Lifetime risk of revision at different ages has been estimated for total knee replacement (TKR). About half of patients needing knee replacement can potentially be treated with either TKR or UKR [13, 41], and the decision as to which is most appropriate depends, in part, on revision rates. UKR is widely considered to be a pre-TKR [30, 35]. The implication of this is that it may be sensible to do a UKR, even though it will be revised at some stage, as it will delay the need for a TKR. As a result many patients, particularly younger ones, are counselled to expect a revision in their lifetime. For patients to decide whether to have a UKR or TKR it would therefore be helpful for them to know their lifetime risk of having a revision if they had a UKR. Currently the lifetime revision risk for UKR is not known.

The evidence from large matched studies is that UKR provides a faster recovery [7], lower morbidity and mortality [19], return to work quicker [18], better function and better satisfaction [20, 38, 41] than TKR. However, UKR are reported in national registries as having a higher revision rate [24–27]. In contrast, multiple long-term series published by those using mobile-bearing medial UKR, with the recommended evidence-based indications and surgical techniques, report revision rates comparable with TKR [1, 22, 23, 31, 33, 39, 42]. The main indication for medial UKR is anteromedial osteoarthritis, in which there is bone-on-bone arthritis medially, full thickness cartilage laterally and functionally normal ligaments [12, 40]. Based on the recommended indications about half of patients requiring knee replacement can be treated with UKR, and surgeons achieving good results tend to use UKR for at least 20% and commonly 50% of their knee replacements. In contrast, most surgeons contributing to the National Joint Registry (NJR) do small numbers of UKR (the commonest number is one per year) and use UKR for less than 10% of their knee replacements, often for early arthritis, which is associated with poor results [17, 21]. Therefore, to reflect what can be achieved with UKR the assessment of lifetime risk of revision should be based on surgeons adhering to the recommended indications and surgical techniques.

In the absence of a large series of UKR followed up until all patients have died, lifetime risk of revision can be calculated based on annual revision rates and death rates in different age groups. Death rates can be determined from national statistics. Annual revision rates should be based on data from well documented patient series. However, these tend to be of limited duration so long term predictions are required, which can be done with parametric survival models. There are different models that may give higher or lower long-term revision rates. It is therefore useful to use a number of different models to assess the accuracy of the long-term predictions.

To help inform patients, surgeons, and health providers, the aim of this study was to estimate lifetime revision risk for patients of different ages undergoing UKR. The hypothesis was that the majority of young patients would undergo revision during their lifetime.

## Materials and methods

### Overview

A state-based Markov model was constructed with transition probabilities for revision informed from a large cohort of consecutive UKR and transition probabilities for mortality informed from the United Kingdom Office for National Statistics. This model was used to estimate the percentage of patients that would be revised over the course of their lifetime. As a sensitivity analysis, assumptions that revision risk would reduce after 90 years of age, and a revision rate from the National Joint Registry were tested.

### Cohort

Between June 1998 and March 2009, 1000 consecutive medial meniscal bearing UKRs were carried out through a minimally invasive approach in 818 patients by two surgeons (CAFD, DWM) who were involved with the design of the prosthesis. All patients met the recommended indications as described by Goodfellow et al. [10]. The pathological diagnosis was bone-on-bone anteromedial osteoarthritis with functionally intact ligaments in 977 knees and spontaneous osteonecrosis of the knee in 23. The mean follow up of this cohort is 10 years (range 5–17). The mean age of the cohort at surgery was 66.6 years (SD 9.6 years, range 33–88; Table 1). 391 knees (332 patients) had minimum 10 years follow up. There were 52 revisions, occurring at a mean age of 66.3 years (SD 8, range 49–80) and mean follow up of 5.6 years (SD 4, range 2 months to 15 years). Twelve of these revisions occurred in patients younger than 60 at intervention. The 10-year survival was 94% (452 at risk, 95% CI 92–96), and the 15-year survival 91% (55 at risk, 95% CI 83–98). 118 patients died (142 knees), 36 patients (42 knees) withdrew from the study because of poor health, and three patients (four knees) were lost to follow up. This patient series has previously been reported in detail [31].

**Table 1:** Cohort demographics

*N*	1000
*N* revisions	52
Age breakdown	
*N* < 60 (%)	242 (24%)
*N* 60– < 75 (%)	552 (55%)
*N* 75+ (%)	206 (21%)
Mean body mass index (SD)	28.5 (5)
*N* female (%)	487 (49%)
Mean follow up in years (range)	10.3 (5–17)

*N* number, *SD* standard deviation

Patients were followed up by research physiotherapists independent of the surgical and clinical teams involved in the care of the patients. Patients were assessed pre-operatively and at 1, 5, 7, 10, 12 and 15 years post-operatively. All patients were contacted to ascertain the incidence of revisions. Revision was defined in the same manner as the National Joint Registry [24], as component removal, exchange or addition. It therefore included insertion of a new bearing for bearing dislocation or washout, addition of a lateral UKR for disease progression or conversion to a total knee replacement (TKR). If a patient had died, information about the status of their knee and any further operation was obtained from primary and secondary care records, and from the patient’s relatives where appropriate.

### Statistical methods

**Fig. 1:**
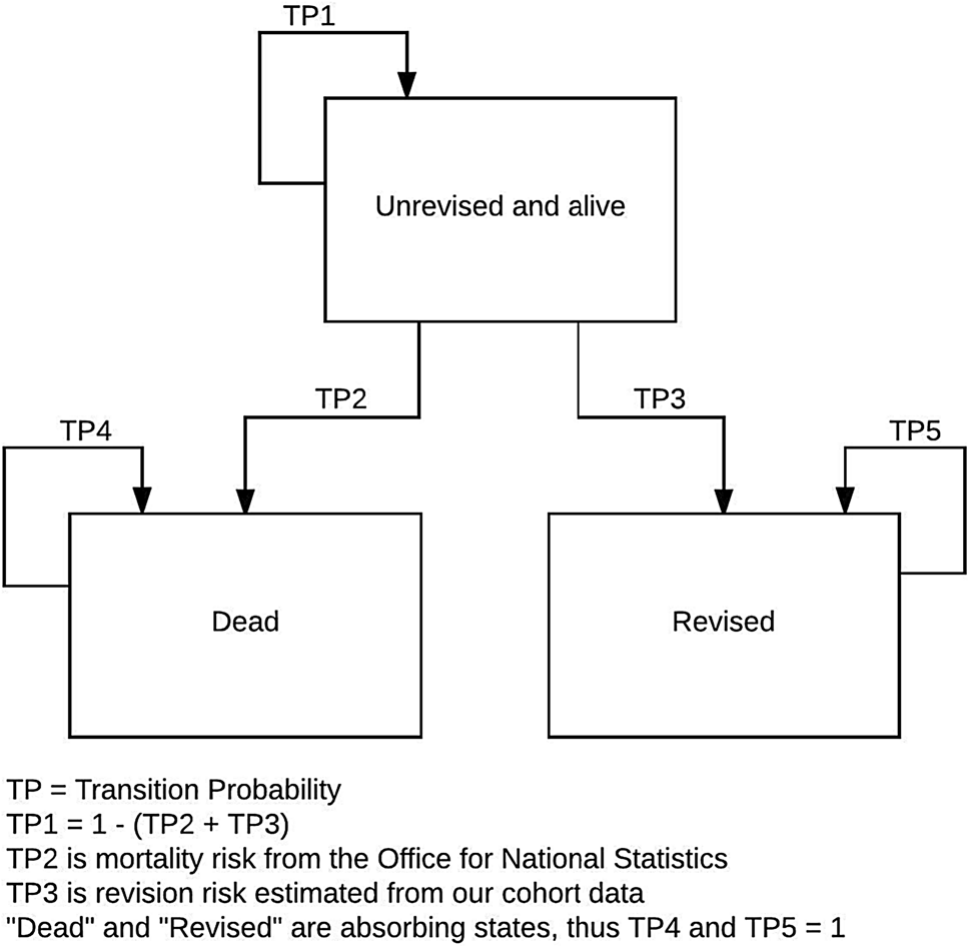
Schematic of Markov model

To extrapolate revision risk, a multi-state Markov model was constructed with three health states: ‘unrevised’, ‘revised’, and ‘dead’ (Fig. 1) [6]. Individuals began as unrevised and then remained so until death or until revised. Such a model reflects the competing risk nature of revision. Transition probabilities to ‘revised’ were informed from parametric regression models fitted to the cohort revision data. A number of alternative distributions for parametric models were fitted separately, these were exponential, Gompertz, Weibull, log normal, log logistic and generalised gamma distribution. The different models allow the revision risk to increase, decrease, or stay the same over time. Akaike’s information criteria (AIC) was calculated for each model as an estimator of the relative quality of each model. Transition probabilities to death were informed from the Office for National Statistics (ONS) National Life Tables [29]. Subjects entered the model at the age of interest following their UKR. Each year, subjects could remain in their current health state, or transition to a new health state. Each of these transitions is represented by arrows in Fig. 1, and had a defined probability of occurring. The simulation was run separately for a cohort of 1000 patients for each age of interest. Confidence intervals were calculated via bootstrapping, with 250 estimations.

Because of uncertainties about the generalisability of this study, as well as the regression models themselves, a sensitivity analysis was performed to assess their effects on the results. The first analysis examined the lifetime risk from the NJR report for mobile bearing UKR. The second analysis tested an assumption that elderly patients are less likely to be offered revision due to comorbidities. For this, the risk of revision was halved after an arbitrarily chosen age of 90. For this analysis, the revision risk was modelled with an exponential model as this had the best fit to the data. The model was validated by estimating incidence rate per 100 component years from 10 years’ worth of predictions, which allowed comparison to observed values from the cohort.

Statistical analyses were performed in R [34] using the survHE package [2].

## Results

**Fig. 2:**
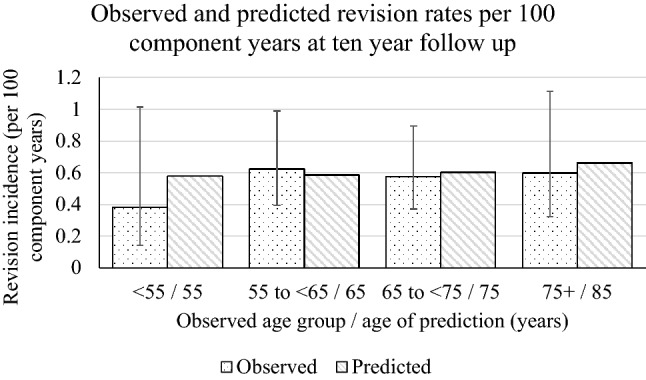
We predicted component time incidence rates after a 10 years simulation to match the mean 10 years observed follow up of this cohort. Predictions were very similar to observed rates

The exponential model had the best fit for estimation of revision probability (AIC 642.8), when compared with the Gompertz (AIC 644.0), Weibull (AIC 644.5), log logistic (AIC 644.7), log normal (AIC 645.7), and generalised gamma (AIC 646.4) models. The exponential model predicted similar component time incidence rates to that observed in the cohort (Fig. 2). Therefore the exponential model was used for the main analysis.

**Fig. 3:**
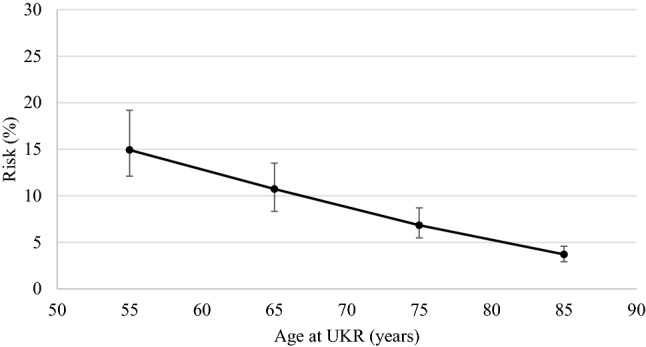
Estimated lifetime revision risk with 95% confidence intervals

**Fig. 4:**
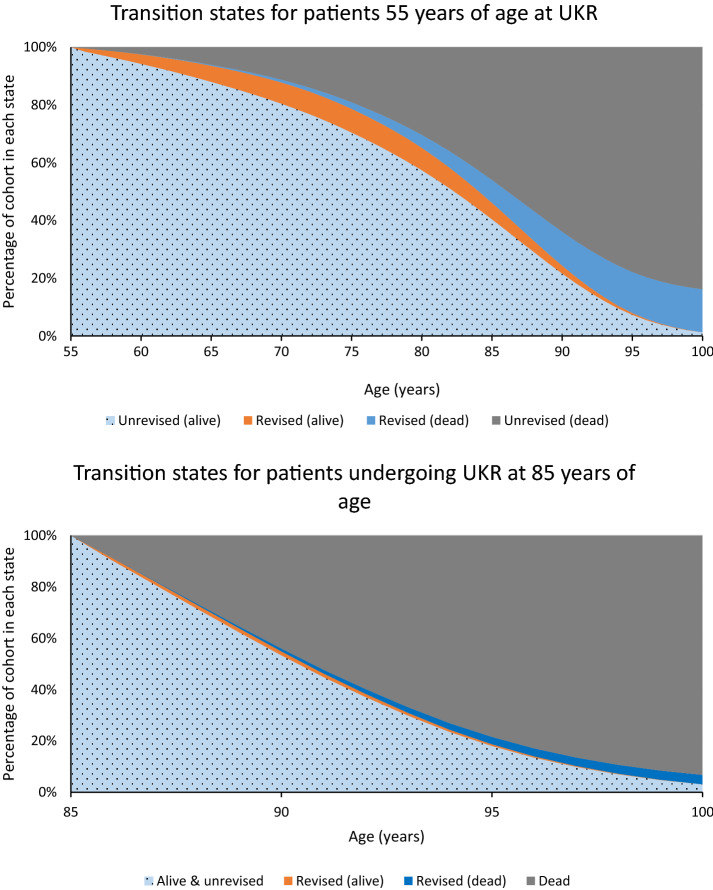
Graphical representation of the competing risk of death and revision in patients of different age groups. Note the difference in scale on the *x*-axis

The estimated lifetime revision risk increases with younger age at operation (Table 2; Fig. 3). The possible outcomes in the years following surgery are shown in Fig. 4. Point estimates for lifetime revision risk from the exponential model were 15% (95% CI 12–19), 11% (8–13), 7% (5–9) and 4% (3–5) for those aged 55, 65, 75 and 85 years at operation respectively. The uncertainty around long-term predictions are reflected in the large confidence intervals in younger patients, which decrease with older age. The different models produced differing estimates (Table 2). The variability between model point estimates from age 55 to 85 respectively was 10% (12–22), 4.5% (10–14), 1.2% (7–8), and 0.1% (3.7–3.8%).

**Table 2:** Lifetime revision risk point estimates from different models (95% confidence interval)

Age at UKR	55	65	75	85
Exponential*	14.9% (12–19%)	10.7% (8–13%)	6.8% (5–9%)	3.7% (3–5%)
Gompertz	22.5% (11–55%)	14.1% (8–28%)	7.8% (5–13%)	3.8% (3–5%)
Weibull	16.2% (12–24%)	11.5% (9–17%)	7.1% (6–11%)	3.8% (3–5%)
Log logistic	15.5% (11–26%)	11.1% (8–17%)	7.0% (5–10%)	3.8% (3–5%)
Log Normal	12.5% (10–19%)	9.6% (7–14%)	6.6% (5–10%)	3.8% (3–5%)
Generalised Gamma	16.0% (12–24%)	11.4% (9–18%)	7.1% (6–10%)	3.8% (3–5%)

*UKR* unicompartmental knee replacement

*The exponential distribution was used for the base-case analysis

The sensitivity analyses had the following results: halving the revision rate from 90 years of age led to estimates of 15%, 10%, 6% and 3% respectively. Increasing the revision rate to that seen in the NJR increased the lifetime revision risk to 25%, 16%, 8% and 3% for those 55, 65, 75 and 85 years at operation respectively.

## Discussion

The most important finding of the study was that the risk of revision of UKR in a patient’s lifetime is low. As was expected, higher rates were found in younger patients. The youngest modelled patient age was 55, and patients of this age were found to have a lifetime revision risk of 15% (CI 12–19). This reduced to a lifetime risk of 4% (CI 3–5) for those of 85 years of age at operation. Surgeons and patients will find these results encouraging, as it is widely expected that patients younger than 60 at operation will almost certainly require revision surgery in their lifetime. Furthermore this study’s hypothesis, based on the perception that the majority of young patients would require a revision during their lifetime, was rejected. The graphs shown in Fig. 4 highlight the various states that the patients may be in as time passes following the surgery, and demonstrate, even in the young how low the risk of revision actually is.

Communication of risk has become increasingly important in patient-centred medicine [9]. As part of the consent process risks and benefits of intervention must be clearly communicated, and there is reasonable evidence to suggest that 5 or 10-year revision risks are difficult for patients to interpret. The James Lind Alliance Priority Setting Partnership, a public-patient involvement group, highlighted that the relationship between timing of joint replacement and best outcome is of great importance to patients. This is a particular concern for young patients, as they are widely expected to outlive their primary joint replacement. Thus, if and when revision surgery may occur becomes a major factor in deciding whether to proceed with surgery. Lifetime revision risk estimates are one way of quantifying the likelihood that they will outlive their prosthesis.

Lifetime revision risk has not previously been estimated for UKR. Burn and colleagues [5] recently looked at 10,000 total knee replacements; and using similar predictive modelling techniques predicted for patients of 50 years of age a 34% lifetime risk of revision, dropping to about 3% for those undergoing TKR at age 80. Further, Evans and colleagues [8] undertook a long term analysis, albeit without age stratification, and using data predominantly from the Finnish arthroplasty registry concluded that the 25 years revision rate was about 18% for TKR, and 30% for UKR. Bayliss and colleagues [3] published detailed lifetime risk estimates for TKR, using different methodology, based on a dataset of over 50,000 patients with a mean follow up of 5 years. Like us, they found that lifetime risk reduces with increasing age and in older patients the lifetime risk of revision was similar with UKR and TKR. Their data would suggest that at age 55 the lifetime risk of revision was about 20% for women and 35% for men. This study found no significant difference between men and women, and found that at 55 the UKR had a lifetime risk of revision of about 15%. This suggests that, providing the UKR is used appropriately, in young women the lifetime risk of revision is similar in UKR and TKR, whereas in young men the lifetime risk may actually be less in UKR. Even if the lifetime risk of UKR and TKR were similar UKR would be advantageous because revision is simpler and is usually a conversion to a TKR [36]. Furthermore UKR provide better PROMs [20], lower morbidity and mortality [19] and a faster recovery [7].

Young age is considered to increase the risk of revision as their relatively higher activity levels place increased mechanical loads and wear on the implant and bone-implant interface. In this context, when compared to TKR, meniscal-bearing UKR has theoretical reasons for reducing the revision rate. The fully congruent bearing has low contact stresses, which minimises linear wear and the risk of the replacement wearing out [11, 37]. It also transmits predominately compressive forces to the bone-implant interfaces, which minimises the risk of loosening [29]. Furthermore, the instrumentation aims to restore ligament tension and function [29], thereby restoring normal knee kinematics [32]. In the observed cohort, the youngest patients have the lowest risk of revision due to disease progression [16]. Although this may seem counterintuitive, it suggests that younger patients may have better quality retained cartilage and bone, and are less likely to fail from lateral osteoarthritis or aseptic loosening, which represent the two main modes of UKR failure. These factors all tend to decrease the revision rate of UKR compared to TKR resulting in the lifetime risk of revision being similar, or perhaps even better in young men who are the most likely to destroy knee replacements.

Many surgeons feel TKR is the best procedure in the elderly, as TKR will not require a revision whereas UKR may. However, as the life expectancy is relatively short in the elderly the lifetime risk of a revision of a UKR is very low (4% at age 85, Fig. 4), and only slightly higher than TKR [3], so other factors need to be considered. In the elderly the rapid recovery, and lower incidence of major medical complications and death are major advantages of UKR [15, 19]. In a matched study of over 100,000 knee replacements, it has been shown that, with UKR compared to TKR, the risk of major medical complications more than halved and, in the first 3 months following surgery, the risk of death is about 50% less, and over 4 years it is about 25% less [19]. In the elderly the background risk of medical problems and death is high, therefore the decreased risk of medical complications and death will far outweigh the slightly increased lifetime risk of revision.

There are limitations of the study: the use of a designer centre series where all patients met established indications to inform revision risk, limits its generalisability. However, in multiple studies it has been shown that provided surgeons use the recommended indications and therefore use UKR for at least 20% of knee replacements they will have similar revision rates to the designer centre [1, 14, 22, 23, 33, 42]. Therefore the results are likely to be generalisable to surgeons using the recommend indications. Further, a recent randomised controlled trial, assessed the results from 68 surgeons across 27 centres in the United Kingdom, and found identical revision rates at 5 years for UKR and TKR [4]. As part of the sensitivity analysis the lifetime revision risk was modelled based on data reported in the NJR, and whilst these estimates were higher, as low volume surgeons who may be using inappropriate indications are included, there was still only a 25% lifetime revision risk in those 55 years of age, which is substantially lower than what many would expect.

Although the dataset was substantially smaller (1000 patients) than Bayliss’, it had double the mean follow up (10 years) and modelled the revision hazard with parametric survival methods, allowing estimation of revision risk beyond the observed data and the construction of a Markov model. Calculated component time incidence rates at 10-year follow up were almost identical estimates to the observed data at all ages except the very young (55 years) (Fig. 2). In the very young the revision rate in the model (0.6% per year) was higher than the observed data (0.4% per year), which suggests that in the young there is a conservative estimate of the revision rate and therefore of the lifetime risk. The results were only slightly influenced by the different parametric models (Table 2), which model the revision rate increasing or decreasing in the long term suggesting that the conclusions are robust.

In conclusion, these estimates of lifetime risk of revision will help patients and surgeons in their decision making process about whether to receive a medial meniscal-bearing UKR. They support the use of UKR as a definitive prosthesis in all age groups provided it is used for the recommended indications.
